# The Role of Carbon Ion Therapy in the Changing Oncology Landscape—A Narrative Review of the Literature and the Decade of Carbon Ion Experience at the Italian National Center for Oncological Hadrontherapy

**DOI:** 10.3390/cancers15205068

**Published:** 2023-10-20

**Authors:** Ester Orlandi, Amelia Barcellini, Barbara Vischioni, Maria Rosaria Fiore, Viviana Vitolo, Alberto Iannalfi, Maria Bonora, Agnieszka Chalaszczyk, Rossana Ingargiola, Giulia Riva, Sara Ronchi, Francesca Valvo, Piero Fossati, Mario Ciocca, Alfredo Mirandola, Silvia Molinelli, Andrea Pella, Guido Baroni, Marco Giuseppe Pullia, Angelica Facoetti, Roberto Orecchia, Lisa Licitra, Gianluca Vago, Sandro Rossi

**Affiliations:** 1 Radiation Oncology Unit, Clinical Department, CNAO National Center for Oncological Hadrontherapy, 27100 Pavia, Italy; 2 Department of Internal Medicine and Medical Therapy, University of Pavia, 27100 Pavia, Italy; 3 Scientific Directorate, CNAO National Center for Oncological Hadrontherapy, 27100 Pavia, Italy; 4 Department of Radiation Oncology, MedAustron Ion Therapy Center, 2700 Wiener Neustadt, Austria; 5 Department for Basic and Translational Oncology and Haematology, Karl Landsteiner University of Health Sciences, 3500 Krems, Austria; 6 Medical Physics Unit, National Center for Oncological Hadrontherapy (CNAO), 27100 Pavia, Italy; 7 Bioengineering Unit, National Center for Oncological Hadrontherapy (CNAO), 27100 Pavia, Italy; 8 Department of Electronics, Information and Bioengineering, Politecnico di Milano, Piazza Leonardo da Vinci 32, 20133 Milan, Italy; 9 Radiobiology Unit, Research and Development Department, CNAO National Center for Oncological Hadrontherapy, 27100 Pavia, Italy; 10 Scientific Directorate, IEO-European Institute of Oncology, IRCCS, 20141 Milan, Italy; 11 Department of Head & Neck Medical Oncology 3, Fondazione IRCCS Istituto Nazionale dei Tumori, 20133 Milan, Italy; 12 Department of Oncology & Haemato-Oncology, University of Milan, 20122 Milan, Italy; 13 Presidency, CNAO National Center for Oncological Hadrontherapy, 27100 Pavia, Italy; 14 School of Pathology, University of Milan, 20122 Milan, Italy; 15 General Directorate, CNAO National Center for Oncological Hadrontherapy, 27100 Pavia, Italy

**Keywords:** carbon ion radiotherapy, particle therapy, radiotherapy, rare tumors

## Abstract

**Simple Summary:**

Carbon ion radiotherapy offers ballistic and radiobiological advantages over conventional photon-based radiotherapy, making it an effective option in case of rare, radioresistant, and difficult-to-treat tumours. The current narrative review aims to critically report the state-of-the-art application of carbon ion radiotherapy in oncological settings, highlighting the clinical activity on carbon ion radiotherapy at the National Center for Oncological Hadrontherapy (CNAO). CNAO is the only Italian facility, and one of four located in Europe using both protons and carbon ions for oncological treatments. Proton and CIRT became fully operational at CNAO starting in 2011 and November 2012, respectively. After an initial ramp-up period, CNAO has progressively honed its clinical, technical, and dosimetric skills and contributed to increasing knowledge on the efficacy, feasibility, and safety of CIRT in selected tumour types, demonstrating the mild rate of toxicities also in case of re-irradiation and tolerance in case of association with systemic treatments.

**Abstract:**

Background: Currently, 13 Asian and European facilities deliver carbon ion radiotherapy (CIRT) for preclinical and clinical activity, and, to date, 55 clinical studies including CIRT for adult and paediatric solid neoplasms have been registered. The National Center for Oncological Hadrontherapy (CNAO) is the only Italian facility able to accelerate both protons and carbon ions for oncological treatment and research. Methods: To summarise and critically evaluate state-of-the-art knowledge on the application of carbon ion radiotherapy in oncological settings, the authors conducted a literature search till December 2022 in the following electronic databases: PubMed, Web of Science, MEDLINE, Google Scholar, and Cochrane. The results of 68 studies are reported using a narrative approach, highlighting CNAO’s clinical activity over the last 10 years of CIRT. Results: The ballistic and radiobiological hallmarks of CIRT make it an effective option in several rare, radioresistant, and difficult-to-treat tumours. CNAO has made a significant contribution to the advancement of knowledge on CIRT delivery in selected tumour types. Conclusions: After an initial ramp-up period, CNAO has progressively honed its clinical, technical, and dosimetric skills. Growing engagement with national and international networks and research groups for complex cancers has led to increasingly targeted patient selection for CIRT and lowered barriers to facility access.

## 1. Introduction

The role of charged particle therapy in cancer treatments continues to grow with protons as the leading modality [1]. Indeed, about 85% of oncological diseases worldwide receiving hadrontherapy are treated with protons, which offer more highly selective energy deposition over conventional photon beam-based radiotherapy (RT) but with a similar biological response [2]. Instead, carbon ion RT (CIRT), another form of charged particle therapy, in addition to the ballistic advantages, is characterised by higher linear energy transfer (LET) than photons or protons, permitting larger energy deposits and a denser ionisation pattern. Furthermore, CIRT has distinctive radiobiological hallmarks exerting 2–3-fold higher relative biological effectiveness (RBE) against intrinsic radioresistant tumours. There are concerns about the cost-effectiveness of charged particle therapy in the radiation oncology community and, considering that CIRT cost is higher than protons, resistance is present even in the proton beam therapy (PBT) community [3].

Currently, 13 of the world’s ion beam facilities provide CIRT for preclinical and clinical activity in Asia and Europe (https://www.ptcog.ch/; accessed on 30 August 2023). Insurance reimbursement differs across the Nations in which CIRT is available. Due to the results obtained by several clinical trials, the Japanese National Health System (NHS) covers the cost for many indications [4]. The Italian NHS currently reimburses particle therapy treatments, with no differences between protons and CIRT for ten indications [5]. In Austria, treatments are supported by social insurance on a case basis and external tumour board review [5].

One of the challenges of CIRT is building solid clinical evidence; for this reason, there is an international effort to achieve this goal. The rarity of the histotypes preferentially treated with CIRT makes it difficult to enrol enough patients for randomised trials, and there is still a lack of results from prospective studies (with level-one evidence). Clinical data have mainly been derived from single-arm prospective studies, dose escalation trials, and small cohort case series reported by the few CIRT facilities operating worldwide.

To date, 55 clinical studies including CIRT for adult and paediatric solid neoplasms have been registered on the www.clinicaltrials.gov (accessed on 30 August 2023) website and three phase III trials are currently recruiting patients to test CIRT versus photon or PBT as standard treatment for unresectable or incompletely resected radioresistant tumours, such as axial chordoma, adenoid cystic carcinoma (ACC), and sarcomas (NCT02838602 and NCT01182779), and for recurrent head and neck (H&N) cancers (NCT04185974).

The National Center for Oncological Hadrontherapy (CNAO) is the only Italian facility, and one of four located in Europe (https://www.ptcog.ch/; accessed on 30 August 2023), using both protons and carbon ions for oncological treatments. After an initial phase of activity (from 2001 to 2010) dedicated to design, construction, beam characterisation, and commissioning, CNAO’s treatment devices (including the synchrotron and all related subsystems) received CE marking in December 2012 and the synchrotron was recognized as a medical device from the Italian NIH.

**Figure 1:**
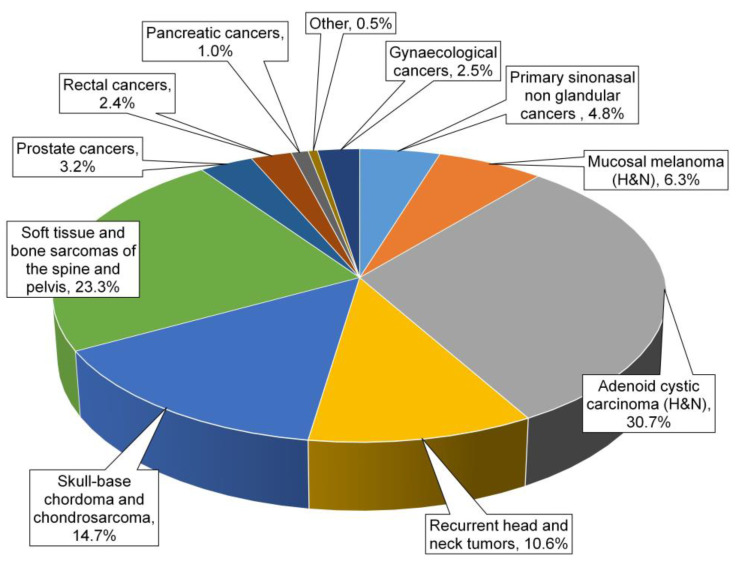
Clinical activities across 10 years of experience with CIRT at CNAO.

Proton and carbon-ion beam therapy became fully operational at CNAO starting in 2011 and November 2012, respectively. By the end of 2022, 4259 patients had been treated, 2238 of whom underwent CIRT (Figure 1). Over these years, we addressed the issue of different RBE models, establishing a methodology for translating prescription doses and dose constraints for organs at risk (OAR) from among clinically adopted models, and creating a framework for international cooperative studies between Europe and Asia [6,7,8].

## 2. Materials and Methods

We conducted a forward literature search in PubMed, Web of Science, MEDLINE, Google Scholar, and Cochrane, until April 2023. Twelve authors carried out the search independently, duplicates were eliminated, and articles were collegially discussed. We then performed a backward literature search on selected articles. In this narrative review, we concisely summarised the most relevant literature clinical results for CIRT (and for PBT in selected histologies) compared with the CNAO’s literature data available so far. The endpoint was to describe the evolution of clinical activities across 20 years of experience at CNAO and in our 10 years with CIRT.

## 3. Results

After the first identification of 145 articles, 111 records underwent first-step screening. Of these, 20 records were excluded because they reported CIRT data regarding tumors that were currently not treated at CNAO or for which there were no studies (pre-clinical, clinical, or dosimetric) ongoing. Ten studies were excluded for being unfit according to the aim of the review or because they reported the same cohort of patients; in these cases, the most recent cohort was considered. An amount of 68 studies were finally analyzed.

### 3.1. Head and Neck (H&N) Cancers

The most common H&N tumors treated at CNAO are primary sinonasal cancers (SNC), mucosal melanomas (MM), adenoid cystic carcinoma (ACC), and relapses of tumors at the edge or in previously irradiated fields.

SNC forms a rare, heterogeneous group of histologies with substantially diverse biological behaviours. CNAO recently reported on only two phase II studies (NCT02099175; NCT02099188) to include CIRT for poor prognosis SNC (including diverse epithelial histological subtypes), adopting a multidisciplinary approach and delivering chemotherapy, surgery, and (photon and/or PBT/CIRT) RT according to disease site and stage [9,10]. Histology-driven induction chemotherapy (IC) was followed by locoregional therapy tailored to IC response in resectable tumours. In particular,

- CIRT was one of the alternative therapeutic options for a mixed beam approach (boost with CIRT of 9–21 GyRBE followed by photons or protons up to 50–60 GyRBE to the large volumes) in the SINTART 1 study [10];
- CIRT was one of the approaches in the SINTART 2 study and can be delivered as a single modality (total dose 68.8 GyRBE with 4.3 GyRBE per fraction in 16 fractions), or in a mixed beam approach (CIRT boost of 21–24 GyRBE with 3 GyRBE per fraction in 7–8 fractions followed by 54 Gy or 54 GyRBE of photons or PBT) according to the volumes to be treated [9].

Concomitant chemoradiation (CT/RT) was limited to good IC responders or primarily non-resectable tumours. Overall, this multimodality treatment proved to be safe, feasible, and effective, especially in IC-responsive patients. Notably, CT was not delivered concomitantly with CIRT. Observed survival rates were similar to those reported for other series, highlighting the need for further research efforts in this rare disease.

Mucosal melanoma (MM) is an ultrarare, aggressive disease. In this scenario, Ramaekers and colleagues [11] reported significantly higher 5-year survival of MM not amenable to surgery after CIRT compared to conventional RT (44% versus 25%; *p*-value 0.007), confirming its safety and efficacy as a sole local treatment modality. Japanese data on unresected patients [12,13,14] suggested considering CIRT as the only alternative to surgery when complete tumour removal is deemed unfeasible or mutilating. Of major concern for the MM is its strong tendency to metastasise early and widely, impacting survival. To the best of our knowledge, the only study to combine CIRT and immune checkpoint inhibitors (ICI) in 10 cases of mucosal MM described an improvement in local control (LC) and progression-free survival (PFS) without severe toxicities when ICI was administered concomitantly or sequentially [15]. To better investigate the safety of this approach, CNAO recently conducted a retrospective analysis [16] in a real-world setting of 33 MM patients (70% H&N, 15% vaginal, 6% anorectal, and 9% skin) treated with sequences of CIRT (median CIRT dose was 65.6 GyRBE for the treatments delivered for primary tumors and 61.5 GyRBE for locally or distant recurrent setting) and ICI. We described 12% grade ≥ 3 acute and 14% grade ≥ 3 adverse effects (AE), corroborating previous reports on AE with CIRT alone (without ICI).

Adenoid cystic carcinoma (ACC), another rare tumour, represents a challenging scenario for radiation oncologists. Its radioresistance, anatomical complexity, and tendency to embrace or intersect radiosensitive structures and follow neural pathways often make it difficult to treat with conventional RT. In recent decades, Japanese centres deemed CIRT to be the sole modality for definitive RT of head and neck ACC (ACCHN), showing good results in terms of LC (5-year LC ranging from 51% to 92%) and acceptable toxicity rates [14,17,18,19]. Considering its neurotropic tendency of spread, the treatment volumes always included the safety perineural pathways. Sulaiman and colleagues [20] retrospectively described the outcome of all ACC patients treated at the four Japanese CIRT facilities between 2003 and 2014. Overall, 289 ACC patients were treated with a median CIRT dose of 64 GyRBE, achieving overall survival (OS), PFS, and LC at 2 years of 94%, 68%, and 88%, respectively. They reported 15% grade ≥ 3 AE, with two grade 5 cases. The Heidelberg Ion-Beam Therapy Centre adopted a different approach, using CIRT as a boost (18–24 GyRBE) to conventional RT (48–56 Gy), achieving 2-year LC and distant PFS of 83% and 81% in nasopharyngeal, 3-year LC of 79% in sinonasal (treated with a radical aim), and 2-year LC of 93% in lacrimal ACC [21,22,23,24]. We recently published preliminary results on 184 consecutive ACCHN treated with a total dose of CIRT ranging between 65.6 and 68.8 GyRBE (4.1–4.3 GyRBE per fraction), with a median follow-up of 45 months (range: 7–90), and 5 year LC and OS of 52.2% and 64.6%, respectively, reporting grade ≥ 3 AE in 19% of patients [25]. In CNAO’s clinical practice, CIRT is delivered in two volume doses: the high-risk clinical target volume (CTV) includes a macroscopic tumour with a margin of 3–5 mm, whereas the low-risk CTV encompasses high-risk CTV plus 2–5 mm of safety margins, the perineural or compartmental spread of the disease. In a subset of this patient cohort, we found that the baseline neutrophil-to-lymphocyte ratio and haemoglobin values appeared to be independent prognostic predictors for clinical outcomes, suggesting that CIRT may dictate the immune response and potentially the outcome [26]. Using the modified microdosimetric kinetic model, we recently recalculated local effect model (LEM) plans for ACC treatment to assess the different impacts on outcome and toxicity [27].

Few studies have described the role of CIRT in relapsed H&N cancers. A prospective randomised clinical trial (NCT04185974) is underway to analyse the safety and efficacy of re-intensity modulated RT (IMRT) compared to re-CIRT for recurrent H&N tumours [28]. Jensen and colleagues reported findings on 52 cases reirradiated with CIRT, with a median dose of 51 GyRBE, achieving 1-year LC of 70.3% and 1-year OS of 81.8% and, despite the high cumulative dose, no higher grade acute reactions. With regards to the higher late toxicities, Jensen et al. reported 3.8% of grade 3 brain necrosis, 3.8% of grade 4 carotid artery haemorrhage, and 5.8% of osteoradionecrosis [29].

Held and coworkers studied 229 patients with recurrent H&N cancer retreated with CIRT, observing local PFS of 24.2 months (95% confidence interval, 19.4–29.0 months), and a median OS of 26.1 months, proving better for ACC compared to SCC tumours. Moreover, the rate of late toxicity grade ≥ 3 was 14.5% [30].

In this scenario, CNAO reported the outcomes of 51 patients with inoperable recurrent salivary gland tumours treated up to a median re-CIRT dose of 60 GyRBE, with 3.0–5.0 GyRBE per fraction [31]. Our data were promising with an actuarial 1- and 2-year PFS of 71.7% and 52.2%, respectively, an estimated actuarial 1- and 2-year OS of 90.2% and 64% [31], and a rate of toxicity comparable with CIRT literature data (grade 3 acute and late, 3.9% and 17.5%, respectively).

### 3.2. Skull Base Chordoma and Chondrosarcoma

Skull base (SB) chordomas pose a challenge for treatment given their proximity to radiosensitive normal tissues and their well-known radioresistance. Mizoe and colleagues [32] reported the results of a dose escalation phase I/II study in 33 patients with SB chordoma at NIRS in Chiba, Japan. A recommended dose escalation of 60.8 GyRBE/16 fractions for four weeks was established due to acceptable toxicity and favourable LC (5- and 10-year LC of 85.1% and 63.8%, respectively). Uhl and coworkers [33] reported on the long-term outcomes of 155 patients treated at the Society for Heavy Ion Research (GSI) in Darmstadt, Germany. With a median follow-up of 72 months, 5- and 10-year LC and OS were 72% and 54%, and 85% and 76%, respectively. In the long-term evaluation (>5 years), approximately 12% and 2% of patients experienced some kind of cranial nerve injury or decreased visual field, respectively. None developed high-grade toxicity involving the inner ear or brainstem.

The CNAO series by Iannalfi and colleagues [34] represents one of the largest prospective outcome analyses yet on 135 SB chordomas, treated with either PBT (70 patients; total dose: 74 GyRBE delivered in 37 fractions) or CIRT (65 patients; total dose: 70.4 GyRBE in 16 fractions) between November 2011 and December 2018. This integrated, customised dual particle (PBT and CIRT) approach took account of multiple features, such as tumour characteristics, toxicity risks, and post-surgery outcomes. After a median follow-up of 49 months (range: 6–87), our group observed 14 (21%) and 8 (11%) local failures in the CIRT and PBT groups, respectively. Five-year LC was 71% for CIRT and 84% for the PBT cohort. The estimated 5-year OS rate was 82% for CIRT and 83% for PBT. Gross tumour volume (GTV), optic pathway and/or brainstem compression, and dose coverage were identified at multivariate analysis as independent prognostic factors of local failure risk. High-rate toxicity grade ≥ 3 was reported in 11% of patients. Very recently, Mattke and colleagues [35] reported on the outcome of 147 patients irradiated with CIRT with a median dose of 66 GyRBE over 4 weeks (111 patients), or PBT with 74 GyRBE over 7 weeks (36 patients), at the Heidelberg Ion Therapy Centre (HIT) in Germany. With a median follow-up time of 49.3 months, 3- and 5-year LC rates were 80% and 61% (PBT), and 80% and 65% (CIRT), respectively. No significant differences were observed between PBT and CIRT in terms of LC, OS, or overall toxicity [35]. An ongoing phase III trial (NCT01182779) on the treatment of SB chordomas is currently comparing 63 GyRBE for CIRT and 72 GyRBE for PBT, with the first endpoint being to evaluate local PFS.

As in the above-reported experience with chordomas, CNAO reported treating SB chondrosarcomas with a tailored approach, delivering PBT or CIRT according to histological characteristics, patient toxicity risk, and GTV volume at baseline MRI. We recently described the outcome of 48 patients treated with PBT (67%) and CIRT (33%) up to a total dose of 70 GyRBE in 35 fractions, and 70.4 GyRBE in 16 fractions, respectively [36]. After a median follow-up time of 38 months, one local failure (2%) was documented with a 3-year LC of 98%. These data are in line with previous PBT and CIRT studies on this setting in which the 3 to 5-year LC ranges from approximately 86% to 100% [35,37,38,39,40,41,42]. One (2%) and four (8%) patients experienced grade 3 acute and late toxicity, respectively. White-matter brain changes were observed in 22 (46%) patients, but only seven needed steroids (grade 2 AE); no patient had grade 3 brain toxicity or grade ≥ 3 complications, demonstrating an excellent safety profile in addition to the promising LC.

### 3.3. Soft Tissue and Bone Sarcomas of the Spine and Pelvis

In advanced malignant pelvic bone sarcomas, often involving nerves and blood vessels, thus generally precluding wide resection, CIRT has proven to be effective. In unresectable chondrosarcomas, CIRT yielded 5-year LC, OS, and disease-free survival (DFS) rates of 53%, 53%, and 34%, respectively [43], with an acceptable toxicity profile [44]. Also in incompletely resected bone and soft tissue pelvic sarcomas, CIRT appeared promising, with 3-year OS, PFS, and LC rates of 83%, 72%, and 92%, respectively, as reported by Demizu and colleagues [45].

CNAO recently published a retrospective experience of 54 consecutive axial bone and soft tissue sarcomas treated with CIRT at a total median dose of 73.6 GyRBE (range = 70.4–76.8), in 16 fractions [46]. In our experience, 2- and 3-year LC rates were 67.4%, and large GTV was predictive of local failure. One-, 2-, and 3-year distant PFS rates were 97.5%, 92.2%, and 92.2%, respectively with a better prognosis for patients undergoing pre-CIRT CT. One- 2- and 3-year OS rates were 87.1%, 75.4%, and 64%, respectively, and OS was significantly influenced by recurrent disease and distant progression. Promising AE results ensued, with only two cases (4%) of grade 3 late neuropathy.

Furthermore, considering dose-averaged LET (LETd)—which steeply rises only in the distal part of the spread-out Bragg peak (SOBP)—we analysed the role of RBE and LET_d_ in the pattern of failures in sacral chordomas. Our results showed that half of the recurrence volumes were in a well-covered high-dose region, but the median LETd values were significantly lower (27 vs. 30 keV/lm) [47], suggesting that combined RBE- and LET-based optimisation could play a key role in improving tumour control. We demonstrated that, in sacral chordomas, high median apparent diffusion coefficient (ADC) values detected at baseline MRI were correlated with CIRT local failure (*p* = 0.003) [48]. Moreover, to identify prognostic and predictive signatures beyond the radiological features in this clinical setting, our group investigated the role of dosiomics in predicting CIRT outcomes. We found that LET_d_ maps showed potential as a risk factor for local recurrence in sacral chordoma, and dosiomics appeared to be the most promising approach against more conventional dose distribution parameters (e.g., DVH-based methods) [49].

Currently, CNAO is involved in the randomised observational SACRO study (NCT02986516), promoted by the Italian Sarcoma Group, comparing upfront surgery with CIRT in patients with resectable sacral chordoma, assessing relapse-free survival as the first endpoint. CIRT could represent an option in preoperative retroperitoneal sarcoma settings to limit toxicity in surrounding healthy tissue for further dose escalation in critical regions at risk for positive margins and in unresectable cases. In the former scenario, a phase II trial (NCT04219202) is ongoing [50], and, in the latter, a retrospective study by Serizawa [51] reported 2- and 5-year LC rates of 77% and 69%, with no grade ≥3 AE after CIRT, at doses ranging between 52.7 and 73.6 GyRBE in 16 fractions.

### 3.4. Prostate Cancer

By virtue of its ballistic characteristics, CIRT has recently been proposed for prostate cancer to minimise AE observed with conventional radiation. The focus is on the benefits of hypofractionation, delivered with CIRT, whose rationale resides in the low *α\β* ratio of prostate adenocarcinoma, and its relative intrinsic radioresistance. The results of three recent phase III non-inferiority randomised trials showed that moderate hypofractionation (2.5 to 3.4 Gy per fraction) has the same efficacy as conventional fractionation photon RT, although long-term efficacy (biochemical and DFS) and tolerability remain to be determined [52]. At CNAO, patients with high-risk prostate cancer are currently treated with 66.4 GyRBE for CIRT delivered in 16 sessions, as derived from the NIRS schedule. We participated in a phase II feasibility study (NCT02672449) of a mixed-beam approach including an anticipated CIRT boost (16.6 GyRBE in 4 fractions), followed by pelvic photon IMRT (up to 50 Gy) for high-risk prostate cancer [53,54], thereby showing promising preliminary feasibility and safety findings. Indeed, no gastrointestinal or genitourinary toxicities were recorded after 1 and 3 months from the whole course of RT.

### 3.5. Rectal Cancer

Radiation is a cornerstone in the treatment of rectal cancer across neoadjuvant, adjuvant, and salvage settings. The Japan Carbon-ion Radiation Oncology Study Group (J-CROS) multicentre study retrospectively analysed the data of 224 RT-naïve patients with local recurrent rectal cancers (LRRC), treated with CIRT at a total dose ranging from 70.4 GyRBE to 73.6 GyRBE [55], achieving promising outcomes. Moreover, 3- and 5-year OS were 73% and 51%, respectively, while 2- and 5-year LC were 93% and 88%, without grade ≥ 4 AE and only 12 cases of late grade 3 AE. In the GUNMA 0801 [56] prospective observational study, 28 LRRC patients not previously treated with conventional RT underwent CIRT at a total dose of 73.6 GyRBE, yielding 3-year OS, LC, and PFS rates of 92%, 86%, and 31%, respectively and two cases of late grade 3 pelvic infections. Phase I and II dose-escalation studies by Yamada and coworkers [57] confirmed these promising results, reporting 5-year LC and OS rates of 88% and 59%, respectively.

Considering its ballistic advantages over conventional RT, CIRT was applied also to LRRC reirradiation. The German experience [58] in 19 cases of CIRT-reirradiation for LRRC led to a median PFS of 20.6 months with no grade ≥ 3 toxicity. Moreover, the Japanese series of salvage CIRT on 77 LRRC patients, after previous pelvic RT, showed 3- and 5-year overall LC rates (in- and out-of-field) of 69% and 62%, respectively, and 3- and 5-year LC for regional recurrences of 85% and 81%, respectively [59]. With a 3- and 5-year OS of 61% and 38%, the toxicity profile seemed safe, with only 21% of late grade 3 AE (pelvic infections, bowel toxicity, skin reactions, pain, and neuropathy). At CNAO we lacked experience with naive-LRRC patients, but managed cases scheduled for reirradiation with CIRT. Our group retrospectively analysed the preliminary data of 14 LRRC patients previously irradiated on the pelvis [60] reporting, after a median follow-up of 18 months, 1- and 2-year LC of 78% and 52% and 1- and 2-year OS of 100% and 76.2%, respectively. In our experience too, toxicity was mild with no grade > 3 AE. Moreover, to study potential predictive markers of CIRT response, we analysed diffusion-weighted imaging MRI data [61]. We found that pretreatment b1000 h-median, b1000 h-interquartile range, and ADC h-kurtosis played a role in predicting the local response of LRRC patients undergoing CIRT.

### 3.6. Pancreatic Cancer

Although in vitro studies provided a biological rationale for using CIRT for pancreatic cancer [62,63], clinical experience of such treatment is limited to phase I and II studies, some of which are still recruiting patients. As yet, no definitive phase III trial data are available; the only randomised trial (NCT03536182) was closed before starting accrual due to the COVID-19 pandemic. In the unresectable setting, the data by J-CROS are encouraging [64] yielding a 2-year OS of 45%, and 2-year DFS of 28%, with no severe AE. In a neoadjuvant study, Shinoto and coworkers [65] assessed the safety and efficacy of preoperative CIRT in 26 cases of resectable pancreatic cancer. Interestingly, said R0 resection rate was achieved in 90% of patients; only two (8%) experienced regional recurrences, and a 5-year OS of 42% was observed. Although one case of late grade 4 portal stenosis was reported, the treatment was well tolerated on the whole. In December 2017 at CNAO, a multicentre phase II trial was initiated to investigate the potential benefits of neoadjuvant chemo-CIRT (6 cycles of FOLFIRINOX followed by a CIRT regimen of 38.4 GyRBE in 8 fractions over 2 weeks), followed by surgery and CT in borderline resectable cancers (NCT03822936) [66]. The study was recently closed due to poor, slow accrual. Data analysis was ongoing at the time of writing. As part of the study, our group critically analysed the challenges of organ motion management [67] including using MRI [68].

### 3.7. Gynaecological Cancers

Studies on CIRT for gynaecological cancers have mainly focused on the most aggressive and poor prognosis tumours. The Japanese group assessed the role of radical CIRT in inoperable endometrial carcinoma (stage: I–III) [69], reporting 5-year LC, PFS, OS, and cause-specific survival rates of 86%, 64%, 68%, and 73%, respectively, with no grade ≥3 acute or late AE. Furthermore, they attributed a predictive role to the total treatment dose needed to achieve a complete response.

Murata and colleagues [70] performed a retrospective long-term follow-up analysis of 37 patients with unresectable gynaecological MM treated with radical CIRT, of which nine cases were post-surgical recurrences. After a median follow-up of 23 months for all patients and 53 months for survivors, 81% of patients experienced tumour disappearance. Two-year LC, OS, and PFS rates were 71%, 53%, and 29%, respectively. Likewise, our preliminary experience with gynaecological MM showed promising results in terms of LC and toxicities [71,72]. In this scenario, we activated a currently recruiting phase II prospective clinical trial (NCT05478876) to assess the efficacy and feasibility of CIRT in unresectable gynaecological MM. At the radiobiological level, we observed a correlation between CIRT dose, activation of dendricity [73], and melanin synthesis in vaginal MM cells, involving also Ca^2+^ signaling [74], paving the way to the further pre-clinical exploration of MM cellular response to CIRT.

CIRT proved to be safe in challenging presentations of cervical carcinomas (i.e., stage IVA with bladder invasion) [75], showing favourable LC, OS, and DFS rates, especially in adenocarcinoma histology, with or without concomitant chemotherapy [76]. Remarkably, salvage surgery for central relapses after a high dose of CIRT seemed to be feasible. Moreover, in human cervical (adeno and squamous) carcinoma cells, Iijima and coworkers [77] demonstrated that CIRT could upregulate PD-L1 expression via phosphorylated Chk1 more than conventional photon RT and in a dose-dependent manner. These findings were also seen in clinical practice by observing that post-CIRT-induced PD-L1 expression (in PD L1 negative patients before CIRT) was significantly associated with improved PFS. Our radiobiology unit is currently testing the effect of CIRT on the modulation of PD-L1 expression on vaginal MM cell lines.

In view of its dosimetric hallmarks, CIRT was also tested on 16 cases of unresectable lymph node recurrences arising in a previous RT field, achieving 3-year OS, LC, and DFS rates of 74%, 94%, and 55%, respectively, with no grade ≥ 3 AE [78]. Considering the Japanese results and CNAO’s preliminary data [79], we recently launched a phase II clinical study on reirradiation of gynaecological recurrences (NCT05457595) with the primary endpoint of LC one year after treatment, defined as the absence of local progression. Oligorecurrent and oligopersistent ovarian cancers appear a promising setting to test CIRT [80].

## 4. Key Points of CNAO’s Learning Curve

Throughout CNAO’s 20-year history and after an unavoidable, intense, and successful learning curve (clinical, physical, and technological), its clinical and dosimetric skills have progressively improved and matured technically.

We dealt with a unique treatment planning process. CNAO beams are fixed, horizontal, and vertical, and they serve three treatment rooms. Robust plan optimization is used, accounting for set-up and range uncertainties and the patient set-up is verified and corrected, daily, through dedicated images (i.e., kV orthogonal images and 3D-CBCT).

To manage the anatomical changes during the treatment we implemented a site-specific adaptive protocol and scheduled re-evaluation CT, based on the tumour location and uncertainties. The solutions to deal with the organ motion include immobilizing the patient with a solid thermoplastic mask, acquiring 4DCT, combining gated dose delivery with rescanning, and performing multiple pre-treatment 4DCTs with multiple breathing phases to assess the reproducibility of respiratory patterns and anatomy before CIRT.

Moreover, CIRT (RBE)-weighted dose (DRBE) significantly depends on the RBE model implemented in the treatment planning system. Our group described a clinically oriented approach to translate LEM-based in mMKM-based prescription doses, reducing the dose distribution difference among CIRT plans [7].

The retrospective dosimetric analysis, based on the dose conversion approach and the clinical outcomes of patients, has been crucial to finding new optimization aims, describing OAR constraints, and consequently ameliorating the treatment quality [27]. To understand the impact of LET on treatment toxicity, we are studying the feasibility of an independent dose and LET calculation system [81]. Considering the impact of simultaneous integrated boost (SIB) in conventional RT, we used an in silico study to evaluate a new CIRT-planning strategy [82] that demonstrated the improvement in conformality and homogeneity of CIRT plans and the reduction in the unintended dose to low-risk volumes, positively impacting toxicity and quality of life. For this reason, we are initiating a prospective study (NCT05733910) on head and neck tumors.

In these 10 years of CIRT, we collected a large number of radiological images, the analysis of which hopefully allows us to find predictive and prognostic markers of clinical outcomes to validate prospectively.

To better understand the radiobiological mechanisms of cell response to CIRT, the Radiobiological Unit is moving from in vitro 2D analysis towards the evaluation of 3D in vitro models and in vivo ones.

Furthermore, CNAO centralizes rare tumours, and for many of them, no guidelines or consensus exists. To enhance the quality of care for this uncommon disease, the efforts in our CIRT decade were focused on creating national and international networking to share the treatment approaches on a case-by-case basis with the possibility of referring pathologic diagnosis to high-expertise centres, surgery to high-skilled groups, and enrol patients in clinical protocols [83].

## 5. Conclusions

**Figure 2:**
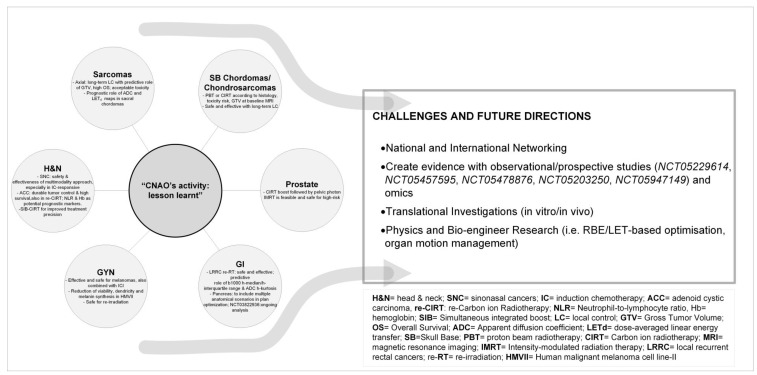
CNAO’s activity “lesson learnt, challenges and future directions”. The figure aims to summarize some of the main results obtained in the 10 years of CIRT at CNAO, as discussed in the dedicated paragraphs of the current review, and to illustrate the upcoming next steps of research.

In our experience, consistent with the international hadrontherapy data, CIRT appears promising in several clinical settings, especially in the case of radioresistant tumors, in which the RBE has proved to be beneficial, and in tumours close to radiosensitive OARs, in which the peculiar ballistic characteristics are advantageous. CNAO has contributed and continues to contribute to increasing knowledge on the efficacy, feasibility, and safety of CIRT in selected tumour types (Figure 1), demonstrating the mild rate of toxicities also in case of re-irradiation and tolerance in case of association with systemic treatments (Figure 2).

Healthcare collaborations between CNAO and other national and international facilities have been established over the years, allowing for more targeted patient selection and management strategies. Clinical networking, along with collaboration with several scientific societies and research groups (AIRO, AIOCC, EPTN, PTCOG, EURACAN, EORTC, MITO, and HitriPlus) has gradually led to a reduction in barriers to facility access. CNAO is fully committed to constantly reporting and updating data gathered from observational studies, and assessing oncological outcomes and toxicity. As summarized in Figure 2, CNAO’s future directions are focused on increasing networking and creating new evidence with the help of its own studies, omics projects as well and participation in national and international research initiatives. To increase the quality of CIRT plans, challenges are also focused on RBE and LET integrations and new tools for managing organ motion. Moreover, the Radiobiology Unit research on the relationship between tumour biology and its interaction with particles is shifting from 2D towards 3D models and in vivo ones. In this complex scenario of effective and safe hadrontherapy delivery, only a complete, comprehensive, mature multidisciplinary approach involving radiation oncologists, medical oncologists, pathologists, radiologists, surgeons, physicists, healthcare institutions, and patients can fully exploit the clinical and organisational benefits of this emerging technology
